# Breaking new ground in heart failure management: novel therapies and future frontiers

**DOI:** 10.3389/fcvm.2025.1643971

**Published:** 2025-08-20

**Authors:** Arbab Khalid, Paulette Rodriguez, Vasiliki Tasouli-Drakou, Abu-Bakr Ahmed, Spencer Thatcher, Jasmine K. Dugal, Aditi Singh

**Affiliations:** ^1^ Department of Internal Medicine, Kirk Kerkorian School of Medicine at the University of Nevada, Las Vegas, NV, United States; ^2^ Kirk Kerkorian School of Medicine at the University of Nevada, Las Vegas, NV, United States

**Keywords:** heart failure, novel therapies, SGLT-2 inhibitors, gene therapy, antifibrotic agents pharmacologic advancements, ARNI, vericiguat, omecamtiv mecarbil

## Abstract

Heart failure (HF) management is advancing in the field of cardiology, driven by the increasing availability of progressive medications. Emerging pharmacological therapies in heart failure management include SGLT-2 inhibitors, ARNI, Vericiguat, and Omecamtiv. Other novel therapies that are changing the scope of HF management include IV Iron therapy and new antifibrotic agents, such as pirfenidone and pamrevlumab. With groundbreaking approaches such as gene and stem cell-based therapies, the possibilities include gene editing and RNA-based therapies. This article contrasts novel HF therapies against previously used therapies, highlighting their mechanism of action, clinical efficiency, and relevant clinical trials. Additionally, the limitations and challenges associated with the emerging therapies will be discussed. Overall, this literature review aims to provide a comprehensive understanding of how these therapies are transforming the management of HF and related cardiomyopathies.

## 1 Introduction

Heart Failure (HF) is characterized by the heart's inability to pump blood efficiently, resulting in an imbalance of myocardial oxygen demand and compromised blood supply to the body, therefore providing insufficient delivery of oxygen and nutrients to tissues. According to the American College of Cardiology (ACC), the American Heart Association (AHA), and the HF Association of America (HFSA), HF is defined as a complex clinical syndrome with symptoms and signs that result from any structural or functional impairment of ventricular filling or ejection of blood (1). HF results in activation of several compensatory mechanisms to maintain cardiac output (CO) and meet systemic oxygen demand. These mechanisms include cardiac remodeling, chronic activation of the sympathetic nervous system (SNS) and renin angiotensin aldosterone system (RAAS), which cause systemic vasoconstriction, increased afterload, and renal hypoperfusion. RAAS also triggers sodium and water retention, which is further potentiated by antidiuretic hormone (ADH) release. Classical symptoms can include dyspnea, fatigue, and signs of volume overload, such as peripheral edema and pulmonary rales. There are many etiologies of HF, but the leading cause is ischemic heart disease due to coronary artery disease (CAD). Non-ischemic causes include valvular causes, hypertension, cardiomyopathies, high-output HF, or tachycardias/arrhythmias.

Per AHA, HF can be staged into Stage A, B, C, and D (1). Patients without symptoms may fall into the Stage A and B categories. Stage A encompasses individuals at risk for HF but without symptoms, structural heart disease, or elevated biomarkers. In Stage B, patients are asymptomatic but show evidence of structural heart disease or increased filling pressures. Stage C involves patients with current or prior symptoms of HF, while stage D is advanced HF that is characterized by multiple attempts to optimize treatment, along with recurrent hospitalizations and symptoms interfering with daily life. For patients in stages C and D, HF can be further classified by left ventricular ejection fraction (LVEF) and New York Heart Association (NYHA) classifications. NYHA classifications range from I to IV depending on a patient's symptoms and functional capacity. NYHA Class I, II, and III individuals have none, slight, or marked limitations in physical activity, respectively, while NYHA Class IV individuals cannot engage in any physical activity without discomfort (2). LVEF classification is crucial as it forms the basis for treatment recommendations and prognosis in HF. HF with preserved ejection fraction (HFpEF) is characterized by an LVEF ≥50%, while a HF with reduced ejection fraction(HFrEF) is defined by an LVEF ≤40% (3). An LVEF between these two thresholds is classified as HF with mildly reduced ejection fraction (HFmrEF). Additionally, previous HFrEF with subsequent LVEF of ≥40% after guideline directed medical therapy get classified as HF with improved ejection fraction (HFimpEF). Overall, diagnosing HF involves a combination of clinical evaluation and non-invasive (echocardiogram, biomarkers, i.e., natriuretic peptide) and invasive (hemodynamic measurement) testing.

HF is associated with substantial morbidity and mortality, increased healthcare expenses due to frequent hospitalizations, and a negative impact on a patient's functional ability and quality of life. HF affects more than 64 million people worldwide (4), with an estimated 6 million affected adults aged 20 years or older in the United States (USA). Over the years, the prevalence of HF has risen due to various factors, with an aging population as the primary contributor and the growing prevalence of comorbid conditions. It is the second most common inpatient diagnosis billed to Medicare among older adults with a high risk of death at one year post-diagnosis. By 2030, both prevalence and health care costs are expected to rise in the USA. The prevalence is projected to rise by 46%, while healthcare costs are forecasted to increase from $30.7 million in 2012 to $53.1 billion (5, 6). Evidence suggests that HFpEF is also becoming more prevalent in the USA, now accounting for at least 50% of the HF population, a significant increase from 24% in 2007%.

Despite advancements, there are notable limitations in current treatments, as many patients on guideline-directed medical therapy (GDMT) do not achieve optimal outcomes and remain severely symptomatic. These patients remain at high risk for frequent HF hospitalization (HFH) and increased cardiovascular mortality (7).

Examples of limitations include inability to tolerate treatment due to adverse effects, costs such as co-pay, and barriers such as prior authorizations that hinder patients from receiving care.

The purpose of this review is to present an overview of the current guidelines on managing HF patients, specifically in HF stages C and D, while also examining emerging therapies that may offer personalized and more targeted treatment options to patients. This could potentially aid in the management of patients with HFpEF, which, according to Shah et al., represents one of the greatest unmet needs in cardiovascular medicine today due to the lack of broadly effective treatments. Other challenges include the limited use of some standard therapies and progression to advanced HF due to adverse effects and contraindications.

## 2 Guideline-directed medical therapy

### 2.1 ACE inhibitors

ACEis are the most commonly indicated medications in the treatment of cardiovascular and renal diseases, including HF, acute coronary syndrome, nephrotic syndrome, diabetes, and hypertension (8). These include lisinopril, captopril, enalapril, ramipril, zofenopril and perindopril. Due to multiple indications, they are one of the most frequently prescribed classes of medications, with lisinopril being the fourth most frequently prescribed drug in 2020 (9)**.** They have been commercially available since 1981, with Enalapril also being the most studied ACEi for HF. Their exact mechanism of action is not completely understood. Although ACEis act on the RAAS, their clinical effects do not correlate directly with plasma renin levels (10). ACEis are competitive inhibitors of ACE and reduce the levels of angiotensin II by diminishing the conversion of ATI to ATII, therefore modulating the RAAS (11). The decreased production of ATII leads to a reduction in the secretion of aldosterone and vasopressin, with the ultimate effect of enhancing natriuresis, lowering sympathetic nerve activity and blood pressure, and preventing remodeling of smooth muscle and cardiac myocytes, therefore preventing hypertrophy. In the context of HF, this ultimately reduces preload and afterload by reducing the systemic vascular resistance and systolic wall stress, which results in increased cardiac output without any increase in heart rate. Also, it is hypothesized that ACEis interfere with the degradation of bradykinin, a peptide that causes vasodilation (10).

ACEis have been shown to improve morbidity, mortality, and hospitalizations in HF patients, while also reducing the incidence of myocardial infarctions (MIs), arrhythmia-induced deaths, and fatal strokes, suggesting that there are multiple mechanisms of benefit, including prevention of ventricular remodeling, anti-ischemic mechanisms, and reduction of neurohormonal activation (12, 13).

Data suggest that there are no differences among available ACEis in their effects on symptoms or survival (14). They are recommended as the first-line medication for HTN and are class 1a and 2b recommendations for HFrEF and HFmrEF patients, respectively. The Co-operative North Scandinavian Enalapril Survival Study (CONSENSUS) and Studies of Left Ventricular Dysfunction (SOLVD) trials showed improvement in mortality when adding Enalapril to the treatment of HF patients (15).

The CONSENSUS trial was a double blind, placebo-controlled randomized trial of 253 patients with NYHA class IV, that demonstrated that treatment with enalapril resulted in a 40% reduction in mortality at six months and a 31% reduction in one year (16). Of note, the CONSENSUS trial did not have an LVEF criterion for entry. The SOLVD trial also significantly demonstrated a reduced mortality of 16% and a 26% reduction in hospitalizations for HF patients on enalapril. Key differences between SOLVD and CONSENSUS are the size of the sample (which was larger in SOLVD), and the criteria of LVEF <35% but excluding overt CHF, mainly NYHA class II-III, in contrast to CONSENSUS, which focused on really severe CHF. Other trials that demonstrate improved mortality include SAVE and VeHFII, which showed a 25% to 28% reduction. Another benefit of ACEis is their link to reduced hospital admissions, as seen in the Assessment of Treatment with Lisinopril and Survival (ATLAS) study. ATLAS study, an international, multicenter, randomized, double blind, parallel group trial compared the effects between high doses (32.5–35 mg daily) and low doses (2.5–5 mg) of lisinopril in 3,164 patients with NYHA class II to IV and LVEF ≤30% (17). The trial found that high-dose lisinopril was more effective compared to low-dose in reducing hospitalizations for HF and all-cause mortality. Besides ACEis' benefit in reducing mortality and HF hospital admissions, there is growing supporting evidence that they have been linked to reducing NYHA classifications and improving exercise capacity. A study that supported these findings was the Perindopril in Elderly People with Chronic HF (PEP-CHF) study, a randomized, double blind, placebo-controlled study that took place in 2006. This study followed 850 participants with HFpEF, aged 70 years or older, who were able to ambulate independently (18),. Although the study did not show differences in mortality or hospitalizations, which was their primary endpoint, it demonstrated improvement in functional capacity with a decrease in NYHA class at year 1.

### 2.2 ARBs

Indications for use of ARBs are similar to those for ACEis. Common ARBs include losartan, candesartan, eprosartan, irbesartan, valsartan, olmesartan and telmisartan. There is now a newer class of medications known as the Angiotensin Receptor–Neprilysin Inhibitors (ARNi), which combines the ARB valsartan and sacubitril, a neprilysin inhibitor. ARBs block the effects of ATII on the AT1 receptor, the latter of whose expression is increased in HF and which is responsible for the maladaptive effects in the remodeling of the heart due to its binding to ATII. In contrast to ACEis, ARBs do not have any effect on bradykinin, but otherwise exert similar effects on blood pressure, renal function, and potassium (13)**.**

Current guidelines place ARBs as a 1b recommendation for pre-HF patients, a Class 2b recommendation for HFmrEF patient, 1a recommendation for HFrEF patients who are unable to tolerate ARNi or ACEis (1). The use of ARBs is furthermore a Class 2b recommendation for HFpEF patients, particularly in patients with LVEF on the lower end of this spectrum.

Clinical trials have demonstrated the efficacy of ARBs in reducing morbidity and mortality in HF, with one of the biggest being the Candesartan in HF: Assessment of Reduction in Mortality and Morbidity (CHARM) program. The CHARM program studied candesartan and consisted of three randomized double blind component trials (CHARM-Alternative, CHARM-Added, and CHARM-Preserved), which ran in parallel. Each trial in the CHARM program was independently designed to determine whether the addition of candesartan to other CHF therapies could reduce the risk of cardiovascular death or hospital admission for CHF (19).

Both CHARM-Alternative and CHARM-Added included patients with LVEF ≤40%, the main difference being that CHARM-Alternative used patients treated with ACEi, while CHARM-Added used patients who could not tolerate ACEi. In contrast, patients in CHARM-preserved had LVEF ≥ 40%. Overall, CHARM-Alternative and CHARM-Added showed that the use of candesartan compared to placebo decreased overall morbidity, mortality, and hospitalization; this reduction ranged from 15%–23%, while CHARM-Preserved did not show a statistically significant reduction. A key limitation in the CHARM program is that it did not include HFpEF patients. Another study that shows the reduced morbidity and mortality is the Valsartan HF Trial (Val-HeFT), which demonstrated a 13.2% reduction compared to placebo (20),. The study design was adding valsartan to conventional treatment, being either ACEi, β-blockers (BB), or spironolactone. Although there was a significant reduction, it was also found that there were *post hoc* observations of an adverse effect on mortality and morbidity in the subgroup receiving valsartan, an ACE inhibitor, and a beta-blocker, raising concern about the potential safety of this specific combination (20).

#### 2.1.1 Cost effectiveness, limitations, and side effects

Several cost-effectiveness analyses consistently found that ACEi and ARB therapy provide high value for patients with chronic HF. A model-based analysis, using generic ACEi costs, found ACEi therapy was of high value (21). Use of ACEi, ACEi + BB, and ACEi + BB + aldosterone antagonist therapies was associated with significant health gains and was cost-saving or highly cost-effective, with the greatest gains in quality-adjusted life year (QALY) occurring when all three medications were provided.

Lisinopril and captopril are the only ACEi that do not have to be activated in the body to be effective. All the other ACEi are prodrugs and require activation (10). Since most of the activation occurs in the liver, a non-prodrug form is preferable in patients with underlying liver issues (22)**.** The Losartan HF Survival Study (ELITE-II) showed that in older HF patients, losartan and captopril had no significant differences in all-cause mortality (11.7% vs. 10.4%), but losartan demonstrated better tolerability (23).

ACEi adverse effects include a dry, non-productive cough that often requires cessation of treatment. The most significant adverse effect is angioedema, which can cause airway compromise. Due to inhibition of kininase and increased bradykinin, ACEi are associated with a higher incidence of non-productive cough and angioedema than angiotensin receptor blockers. For both ACEi and ARBs, limitations include giving caution to patients with low systemic blood pressures <100 mmHg, renal insufficiency, or elevated serum potassium (>5.0 mEq/L), also abrupt withdrawal of ACE inhibition can lead to clinical deterioration and should be avoided (1). There is a higher risk of worsening renal function in patients with already renal insufficiency and renal artery stenosis with ACEi. In susceptible groups, monitoring of renal function and electrolytes is recommended.

### 2.2 Beta-blockers

BBs are a highly varied class of drugs. Beta receptors exist in three different forms: Beta-1 (B1), Beta-2(B2) and Beta-3 (B3). B1 receptors are primarily located in the heart and mediate cardiac activity. B2 receptors have a more diverse function due to being located in multiple organs. Some of the functions include vasodilation, bronchodilation, and controlling metabolism, while B3 receptors are mainly in charge of lipolysis and bladder relaxation. In HF, in addition to RAAS activation, another compensatory mechanism is involved, which is the activation of the sympathetic nervous system(SNS). Collectively, this is referred to as neurohormonal activation (24). The increased SNS activation leads to B1 adrenoceptor-mediated increase in renin, therefore activating RAAS. Neurohormonal activation, in turn, increases norepinephrine, angiotensin II, and aldosterone. These effects can be counteracted by BBs. Chronically increased SNS activation significantly contributes to the progression of left ventricular hypertrophy to myocardial dysfunction due to increased myocardial energy, oxygen demand, and oxidative stress (25). BBs are widely used. They are one of the four pillars of GDMT; specifically, bisoprolol, carvedilol, and metoprolol succinate are the commonly chosen agents. They differ in their peripheral vascular effects and selectivity for adrenergic receptors. Carvedilol is a non-selective drug that can also interact with alpha receptors, while bisoprolol and metoprolol succinate are β1-selective agents.

Non-selective BBs block both beta-1 and beta-2 adrenergic receptors, along with alpha-1 receptors. Blocking alpha 1 receptors yields vasodilation, thus reducing afterload and enhancing cardiac output. Non-selective BBs also have antioxidant effects and help reduce oxidative stress caused by catecholamines, which may play a role in their effectiveness for treating HF. A meta-analysis by DiNicolantonio et al. that compared carvedilol to other beta-1 selective BBs demonstrated that carvedilol appears to offer superior mortality benefits in systolic HF and acute MI (26).

Per AHA 2022 guidelines, BBs are Class 1b for pre-HF, Class 2b for HFimpEF/HFmrEF, and 1a for HFrEF. Multiple studies showed that the use of BBs, in particular HFrEF, reduced mortality and hospitalizations. The Metoprolol CR/XL Randomized Intervention Trial in HF (MERIT-HF) demonstrated a 34% reduction in mortality among patients treated with metoprolol succinate compared to placebo (27). Similarly, the Carvedilol Prospective Randomized Cumulative Survival (COPERNICUS) trial showed a 38% reduction in mortality and a 31% reduction in the risk of death or hospitalization for HF with carvedilol (28).

Limitations in treatment include contraindications in severe bradycardia, second or third degree heart block without a pacemaker, overt cardiogenic shock, hypotension, and severe bronchospastic disease. Other limitations include the use of BBs with transthyretin amyloid cardiomyopathy, as it can worsen HF symptoms because these patients rely on heart rate response to maintain cardiac output (1). Other limitations include insulin-dependent diabetes due to masking of hypoglycemic symptoms and peripheral arterial disease, as non-selective BBs may worsen symptoms. The most common cause of non-compliance with BBs is adverse effects. Per Kalra et al, the most common adverse events associated with discontinuation were dyspnea, fatigue, and dizziness, occurring at rates of 98, 53, and 49 per 1000 patient-years, respectively (29).

### 2.3 Mineralocorticoid receptor antagonists

Mineralocorticoid Receptor Antagonists (MRAs) like Spironolactone and Eplerenone are recognized as effective agents for HF management (30). MRAs' mechanism of action includes blocking mineralocorticoid receptors (MR), which are found in the kidneys or heart, and blood vessels. Elevated aldosterone levels contribute to fluid retention in HF and lead to hypertension and myocardial fibrosis (31). MRAs counteract these harmful effects by inhibiting aldosterone binding to its receptor, which reduces sodium retention, vascular remodeling, and myocardial fibrosis to improve cardiac function. MRAs exert anti-inflammatory and anti-fibrotic effects in myocardial tissue are crucial in mitigating pathophysiological processes of HF (32).

Clinical trials such as the RALES trial have demonstrated the life-saving efficacy of Spironolactone in patients with severe HFrEF. It shows a 30% reduction in mortality, with a significant decrease in hospitalization rates. Eplerenone is a more selective MRA with fewer endocrine side effects and has also shown efficacy in HF patients post-MI in the EMPHASIS-HF trial, demonstrating reduced cardiovascular mortality and hospitalization (33), These agents have become integral in HF management for patients with moderate to severe HFrEF and often in combination with RAAS inhibitors like ACEis or ARBs. MRAs have some limitations and risk factors, including hyperkalemia, which is the most prominent side effect, more frequently found among patients with renal impairment, as MRAs decrease potassium excretion in the kidneys. Renal dysfunction is another concern necessitating close monitoring of renal function and electrolytes during treatment. Patient adherence can be an issue due to the side effects and the need for regular blood tests, which can deter long-term use among older adults or those with comorbidities (34).

Finerenone, another MRA, deserves a special mention. An analysis of three different trials (FIDELIO-DKD, FIGARO-DKD and FINEARTS-HF) in patients with HF, CKD and type 2 diabetes found that finerenone compared to placebo reduced the risk of hospitalization from heart failure, the risks for deaths of any cause, cardiovascular events and composite kidney outcomes (35). Compared to Spironolactone and Eplerenone, it has been found to have a stronger mineralocorticoid receptor binding potential, along with anti-inflammatory and anti-fibrotic effects (36). Moreover, in the the Phase II ARTS trial, it was found to be similarly effective to spironolactone, with fewer ewer episodes of worsening kidney function and hyperkalemia (37). It will thus not be surprising to see a rise in the use of finerenone given its demonstrated effective treatment for kidney and cardiovascular protection (38).

### 2.4 ARNI

ARNIs such as Sacubitril/Valsartan represent a novel class in HF treatment, and this combination drug inhibits two critical pathways: the AT1 and neprilysin. Sacubitril inhibits neprilysin, which is an enzyme responsible for breaking down natriuretic peptides (e.g., BNP and ANP), and it has an important function in fluid overload and promoting vasodilation. It prevents degradation of these peptides, so neprilysin inhibition increases their beneficial effects. Valsartan is an ARB, which means it prevents the harmful effects of angiotensin II, which include vasoconstriction, sodium retention, and myocardial remodeling (39),.

For instance, the PARADIGM-HF trial is a landmark study that demonstrated that Sacubitril/Valsartan reduced the risk of death from cardiovascular causes by 20% and the risk of HF hospitalization by 21% compared to enalapril, which is an ACEi. Results were consistent across various subgroups which solidify efficacy of ARNI in reducing both mortality and morbidity in HFrEF patients and beneficial effects extend beyond symptom relief with evidence showing improvement in quality of life and exercise capacity (40),. Most current trials have expanded the potential role of ARNI beyond HFrEF while exploring its impact on HFpEF. Early data suggest that ARNI may help reduce hospitalizations and improve clinical outcomes in HFpEF patients, but more definitive data are awaited. Sacubitril and Valsartan have been explored in chronic kidney disease (CKD) and diabetic nephropathy, where their use has reported promising outcomes in reducing albuminuria and preserving renal function. ARNI therapy has drawbacks, such as some patients still find the expense to be exorbitant, and renal side effects, specifically, hypotension and hyperkalemia, are frequent problems. When prescribing sacubitril/valsartan, care must be taken because it may interfere with other medications, including potassium-sparing diuretics and ACEi, and ARNI usage may not be appropriate for a sizable percentage of patients because of pre-existing angioedema or renal impairment (41).

### 2.5 Diuretics

Diuretics, such as Furosemide and Torsemide, are essential in the management of HF, primarily for controlling fluid overload. These drugs work by inhibiting sodium and chloride reabsorption in the renal tubules, leading to increased urine output. In HF, excess fluid accumulation contributes to pulmonary congestion, edema, and symptoms such as shortness of breath, which diuretics alleviate by promoting the excretion of sodium and water, thereby reducing preload and improving hemodynamics. Diuretics are particularly critical in the acute decompensated phase of HF, where rapid fluid removal can improve symptoms and reduce hospitalizations. Furosemide, as a loop diuretic, is the most commonly used in hospitalized patients due to its potent action and rapid onset. However, chronic use of diuretics in stable patients with HF is also common, as it helps manage persistent symptoms of fluid retention (42).

Recent advancements in diuretic therapy have focused on optimizing dosing schedules, combination therapies, and minimizing side effects. Fixed-dose combinations, such as those combining loop diuretics with thiazide diuretics, are gaining popularity, as they can potentiate the diuretic effect and prevent the development of diuretic resistance in patients with advanced HF. Research has also explored alternative agents, like the potassium-sparing diuretic Spironolactone, to mitigate electrolyte imbalances commonly seen with long-term diuretic use (43). Despite their usefulness, diuretics have limitations. Electrolyte imbalances, particularly hypokalemia, hyponatremia, and hypomagnesemia, are common side effects that can lead to arrhythmias, muscle cramps, and weakness. Renal dysfunction, often exacerbated by high doses of diuretics, is another concern. Furthermore, the potential for diuretic resistance in advanced stages of HF, where the kidney's ability to excrete sodium diminishes, presents a challenge. Patients may also develop symptomatic hypotension, increasing the risk of falls and further complicating HF management (44).

### 2.6 Sodium-glucose cotransporter 2 inhibitor (SGLT-2i)

SGLT-2is, such as Empagliflozin and Dapagliflozin, have emerged as a groundbreaking treatment for HF, especially in patients with type 2 diabetes and CKD (45). These agents work by inhibiting the sodium-glucose cotransporter 2 (SGLT-2) in the proximal renal tubules, leading to increased urinary glucose excretion. While initially developed for diabetes management, SGLT-2is have shown remarkable cardiovascular benefits, including reductions in HF hospitalizations, mortality, and renal decline (46). The EMPEROR-Reduced and DAPA-HF trials have provided robust evidence for the use of SGLT-2is in HFrEF, demonstrating significant reductions in the risk of hospitalization for HF and cardiovascular death. These benefits are consistent even in patients without diabetes, highlighting the broader applicability of these agents in HF management. Additionally, the EMPEROR-Preserved trial is exploring their efficacy in HFpEF, with early data showing promising reductions in hospitalizations and clinical worsening of HF (46).

Ongoing research is investigating the potential of SGLT-2is in CKD, with studies suggesting that they may slow the progression of renal dysfunction in HF patients, especially those with diabetic nephropathy (47). Furthermore, combination therapies that include SGLT-2is are being explored, particularly in patients with both HF and CKD, to improve outcomes across both domains (47). However, SGLT-2is are not without their limitations. Genitourinary infections, including urinary tract infections and genital mycotic infections, are common side effects. Volume depletion, leading to hypotension, and the risk of diabetic ketoacidosis in susceptible patients are also significant concerns. Additionally, while these medications are beneficial in HFrEF, their role in HFpEF is still being evaluated, and the cost remains prohibitive in some regions, limiting accessibility.

## 3 Novel therapies

### 3.1 Vericiguat

**Figure 1:**
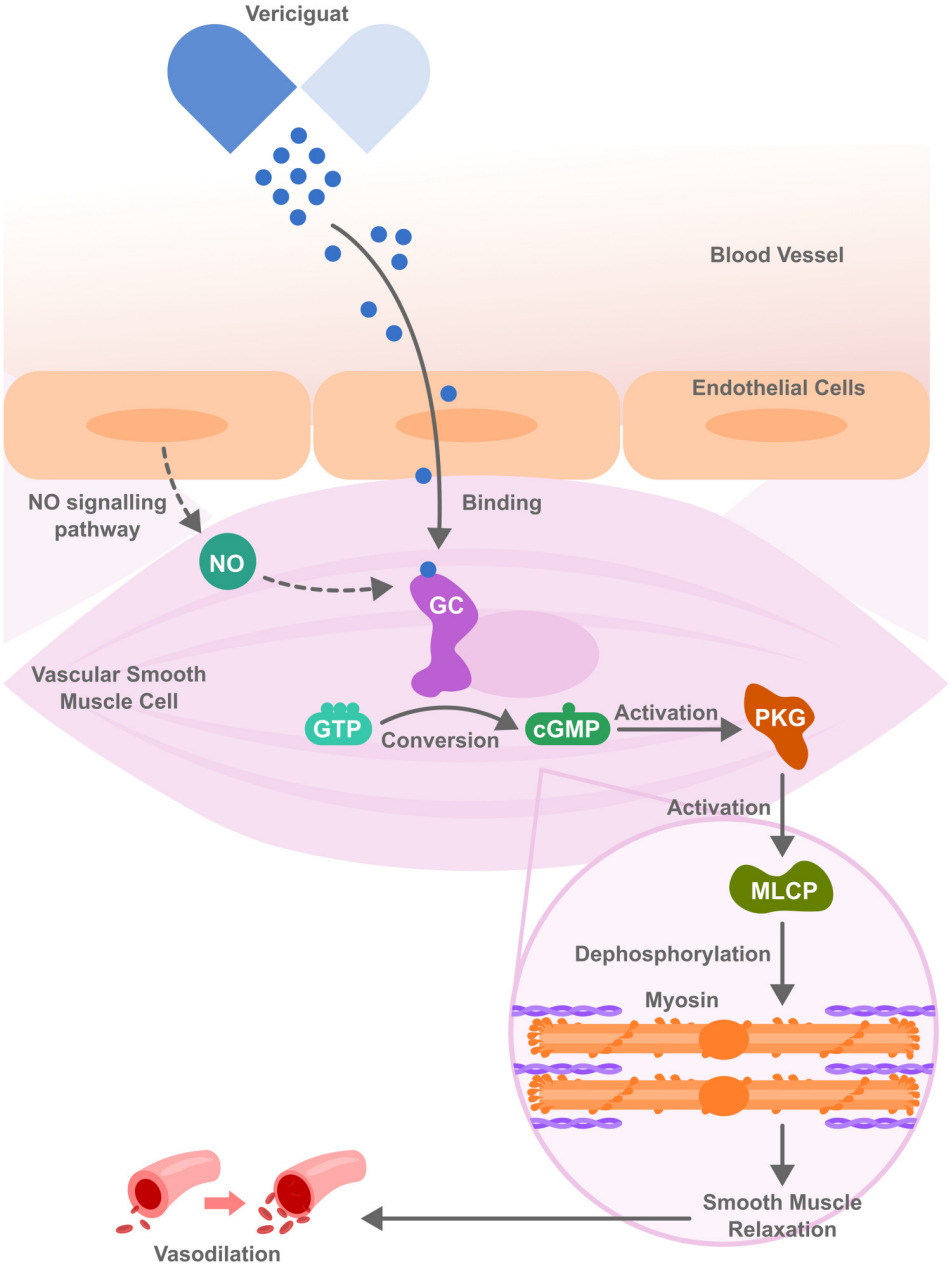
Vericiguat molecular mechanism of action. It acts by stimulating guanylate cyclase (sGC), both independently and in synergy with nitric oxide (NO) thereby activating cyclic guanosine monophosphate (cGMP) and further activating protein kinase G (PKG). PKG then activates myosin light chain phosphatase (MLCP), triggering a signaling cascade that leads to muscle relaxation and vasodilation, ultimately improving blood flow and reducing cardiac workload.

Vericiguat is a novel soluble guanylate cyclase (sGC) stimulator that enhances the nitric oxide (NO)–sGC–cyclic guanosine monophosphate (GMP) signaling pathway, as depicted in Figure 1. Stimulating sGC increases the production of cyclic GMP, promoting vasodilation, enhancing endothelial function, reducing cardiac remodeling to improve cardiac function, particularly in patients with HFrEF (119). The Vericiguat Global Study in Subjects with HF with Reduced Ejection Fraction (VICTORIA) trial was a randomized, double blind, parallel study design trial performed to evaluate the effect of vericiguat compared to placebo in patients with chronic HFrEF. Specifically, the effect of vericiguat in those with HFrEF was studied in patients who were recently hospitalized or had received intravenous diuretic treatment (119). Over 5000 patients with chronic HFrEF received a target dose of Vericiguat 10 mg once daily or placebo, both in addition to GDMT (119). It was found that in patients with high-risk heart failure, the incidence of death from cardiovascular causes or hospitalization for HF was reduced among the patients who received vericiguat compared to those in the placebo group (119).

Potential side effects of vericiguat include dizziness, headache, and hypotension, which are attributable to nitric oxide-related vasodilation and require careful patient selection and monitoring. It should also be avoided in patients taking medications that also affect this pathway, such as long-acting nitrates, PDE-5 inhibitors (e.g., sildenafil), or other soluble guanylate cyclase inhibitors, as concomitant use of both can result in a higher risk of hypotension and syncope (48). A significant limitation can arise from tolerance development with long-term use, which may diminish its efficacy (49). To note, vericiguat carries a black box warning for embryo-fetal toxicity and is contraindicated in pregnancy. Women with childbearing potential should be on contraception forms during treatment, and it's advisable to continue for even up to 1 month after stopping treatment. The relevance of sGC-cGMP signaling pathway to HfpEF pathophysiology remains unclear. Barriers to implementation in HFpEF include the absence of proven clinical benefit, limiting Vericiguat's approval and evidence base to HFrEF as seen in the VICTORIA trial.

### 3.2 Omecamtiv mecarbil

**Figure 2:**
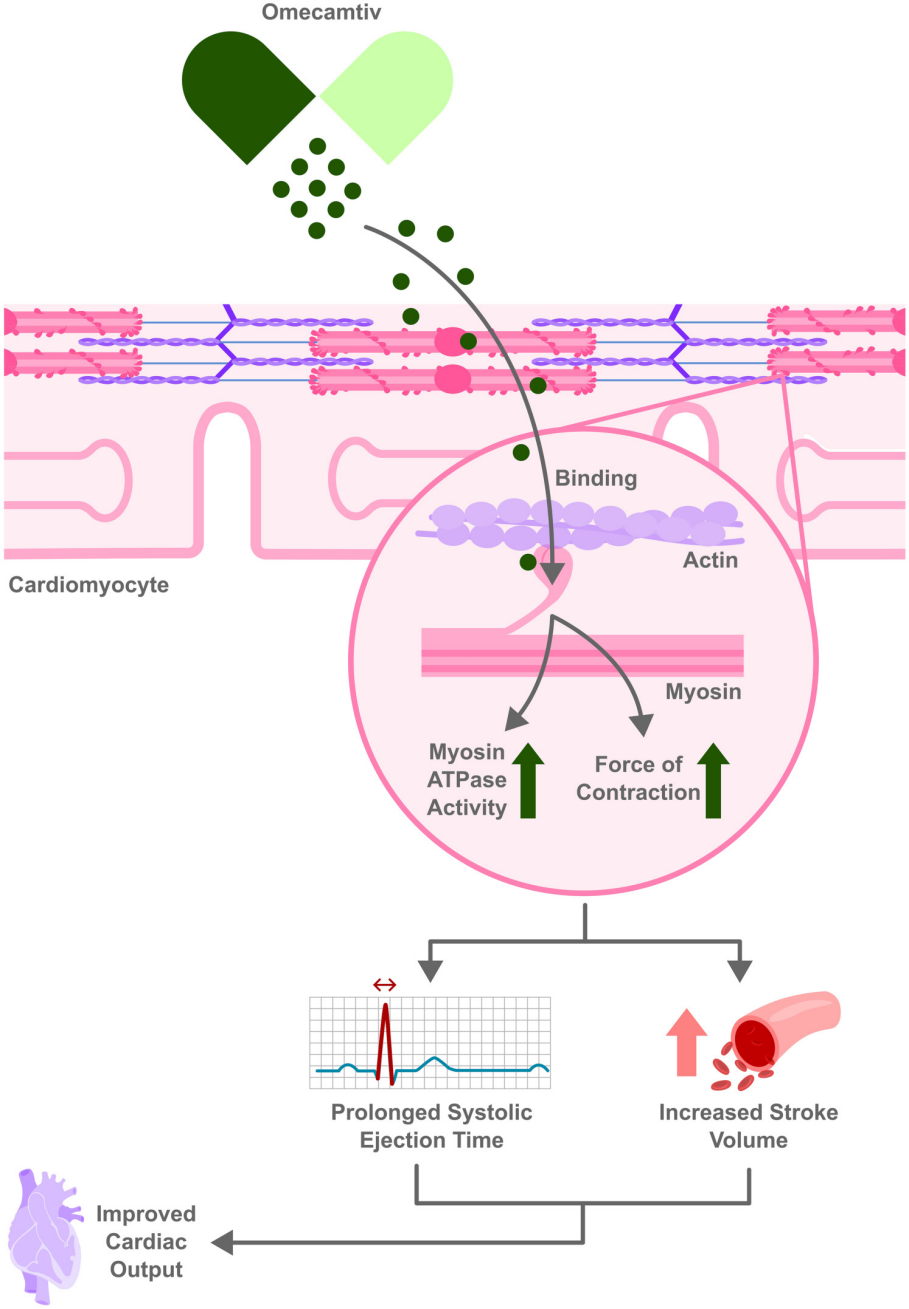
Omecamtiv Mecarbil enhances heart function by binding to myosin increasing actin–myosin interaction without raising intracellular calcium. This leads to prolonged systolic ejection time and increased stroke volume, leading to improved cardiac output.

Omecamtiv mecarbil is a first-in-class cardiac myosin activator (sometimes termed a “myotrope”). Unlike traditional inotropes, it does not increase intracellular calcium or cAMP; instead, it binds to the myosin S1 domain and stabilizes the actin–myosin interaction (50). This prolongs systole and augments stroke volume, thereby improving contractility without markedly raising myocardial oxygen consumption or precipitating arrhythmias (51).This mechanism is illustrated in Figure 2. Preclinical and early clinical studies showed that omecamtiv increases ejection fraction, reduces left ventricular end-diastolic volume, and lowers natriuretic peptide levels, indicating favorable ventricular remodeling (50). These unique mechanistic properties aim to enhance cardiac performance in systolic HF while avoiding the mortality risks seen with older inotropes.

The 2021 GALACTIC-HF trial evaluating omecamtiv among 8,256 patients with HFrEF (LVEF ≤35%) found modestly but significantly reduced the composite primary endpoint of first HF event or cardiovascular death (37.0% vs. 39.1% with placebo; HR 0.92, 95% CI 0.86–0.99; *p* = 0.03) (52). This effect was driven mainly by fewer HF hospitalizations, as there was no significant reduction in cardiovascular mortality (≈19.5% in both groups) (52). Quality-of-life measures (e.g., KCCQ score) did not significantly differ between omecamtiv and placebo. Notably, *post-hoc* analyses suggested greater relative benefit in patients with very low ejection fraction (e.g., EF < 30%) (53).

Another study, METEORIC-HF in 2022, assessed the impact of omecamtiv on functional capacity. In 276 well-treated HFrEF patients, omecamtiv failed to improve exercise capacity over 20 weeks. There was no difference in peak oxygen uptake (VO₂ peak) or other cardiopulmonary exercise test metrics between omecamtiv and placebo. Daily physical activity levels and patient-reported functional capacity also remained unchanged, underscoring that improved clinical outcomes with omecamtiv may not translate into improved exercise tolerance (54).

Omecamtiv mecarbil has been generally well tolerated in trials. In GALACTIC-HF, rates of arrhythmias and ischemic adverse events were similar to placebo (52). There was a small dose-dependent increase in troponin I levels with omecamtiv (median +4 ng/L), but no excess in clinically evident myocardial ischemia (52). Occasional liver and kidney function changes, as well as a transient white blood cell count increase, were also seen. Unlike beta-agonist inotropes, omecamtiv does not significantly raise heart rate or blood pressure. Electrolyte abnormalities (e.g., hypokalemia) and pro-arrhythmic effects seen with some inotropes have not been a major issue (53). That said, careful dose titration is required due to its narrow therapeutic index and use of pharmacokinetic-guided dosing in trials (52).

The clinical benefit of omecamtiv is viewed as modest. The absence of a mortality benefit or quality-of-life improvement in the pivotal trial has raised questions about its overall impact. In 2022, an FDA advisory committee voted against approval of omecamtiv for HFrEF (8–3 vote), citing the small absolute risk reduction in GALACTIC-HF (2.1% fewer events over ∼22 months) and lack of symptomatic improvement (53). The FDA followed this recommendation and did not approve the drug (55). Some experts argue omecamtiv might be useful in niche populations, such as those with very low EF or who cannot tolerate other therapies (53), given that it has shown modest benefit in those with an LVEF below 28%. In this subgroup, omecamtiv was associated with a reduction in heart failure-related hospitalizations, improvement in left ventricular structure and function, and enhanced systolic performance. Despite reducing hospitalizations it did not show a significant mortality benefit, making omecamtiv more of a complementary therapy rather than a stand-alone treatment. Ongoing analyses and potential future trials may clarify if specific subgroups derive meaningful benefit or if combination with other therapies could enhance its efficacy. For now, omecamtiv mecarbil represents a novel mechanism with proof of concept for improving pump function, but with limited translation into hard outcomes.

Despite these advantages, there are several barriers to omecamtiv's widespread clinical use. One concern is that excessive omecamtiv concentration can paradoxically decrease rather than increase cardiac contractility, as seen in one study with canine cardiomyocytes. Another is the optimal dosing in humans, which remains uncertain. Additional research is needed to thoroughly evaluate and address issues such as limited diastolic time, particularly in higher heart rates or HFpEF patients (55, 56).

### 3.3 Intravenous iron in HF

Iron is essential not only for hemoglobin synthesis but also for cardiomyocyte enzymes that are central to oxidative phosphorylation, the primary source of energy for cardiac contractility. Iron acts as a cofactor mainly integrating into heme proteins and Iron-sulfur(Fe-S) clusters that are essential for mitochondrial oxidative metabolism and oxygen handling. Cardiomyocytes have several routes for iron entry but only one pathway for iron export making them vulnerable to iron overload conditions. They primarily acquire iron through transferrin receptor 1 (TfR1)-mediated endocytosis of transferrin-bound iron. Alternative pathways include non-transferrin bound iron (NTBI) entry via divalent metal transporter 1 (DMT1), L-type and T type calcium channels and Zinc transporters (ZIP8/14).

Iron deficiency (ID) is a common comorbidity in HF, affecting roughly 50% of patients with HFrEF. It occurs even in the absence of anemia and is associated with diminished exercise capacity, poorer quality of life, and higher risks of hospitalization and mortality (57). In HF, ID contributes to impaired skeletal muscle function and diminished myocardial energetics, exacerbating fatigue and exercise intolerance (58). Mechanisms in which ID impacts HF vary for example in HFrEF ID contributes to myocardial dysfunction by reducing the heart's energy consumption while HFpEF patient exhibit ventricular stiffness and diastolic dysfunction exacerbated by inflammation and ID.

IV iron therapy, usually with ferric carboxymaltose (FCM) or ferric derisomaltose, can replenish iron stores more rapidly and fully than oral iron, leading to improvements in hematinic indices and tissue iron availability. This can improve aerobic capacity and symptoms. Over the past decade, multiple trials have tested IV iron in HFrEF, showing benefits in symptoms and functional status, even in patients without significant anemia (58).

The FAIR-HF and CONFIRM-HF trials demonstrated that IV FCM significantly improved NYHA class, six-minute walk distance, and patient global assessment scores compared to placebo**.** In FAIR-HF (*n* = 459), 50% of patients on FCM reported marked or moderate improvement in symptoms vs. 30% on placebo (a 20% absolute increase), and six-minute walk distance increased by ∼35 meters (58). NYHA class also improved with 47% of FCM-treated patients improving by at least one class vs. 30% with placebo. CONFIRM-HF (*n* = 304) extended follow-up to 52 weeks, confirming sustained benefits: patients receiving IV iron had continued improvement in exercise capacity and a significant reduction in risk of HF hospitalizations compared to placebo (58). These trials solidified that treating iron deficiency (ferritin <100 µg/L or 100–299 with transferrin saturation <20%) leads to better exercise tolerance and quality of life in HFrEF, independent of anemia status (59).

The AFFIRM-AHF trial extended these findings to recently hospitalized patients with acute decompensated HF. This trial enrolled 1,132 patients hospitalized for acute decompensated HF who were iron-deficient, and treated them with IV FCM or placebo shortly after stabilization and at discharge. Over 52 weeks, IV iron therapy led to a 26% relative reduction in total HF hospitalizations (FCM group 48.9% vs. 53.5% in placebo experienced ≥1 HF rehospitalization; *p* = 0.013) (60). The composite primary endpoint (total HF hospitalizations plus cardiovascular death) was numerically lower with IV iron (52.5% vs. 67.6% of patients had an event), but this did not reach conventional significance (*p* = 0.059). Importantly, there was no difference in cardiovascular mortality alone (≈14% in both groups) (60). AFFIRM-AHF suggests that treating iron deficiency in the acute HF setting can reduce recurrent admissions, although the trial was underpowered to confirm a mortality benefit.

The IRONMAN trial (UK, *n* = 1,137) was a pragmatic, open-label study examining long-term IV iron (ferric derisomaltose) in chronic HFrEF. Over a median 2.7-year follow-up, the primary endpoint of recurrent HF hospitalizations and CV death was lower in the IV iron group, but with borderline significance (rate ratio 0.82, 95% CI 0.66–1.02; *p* = 0.07) (59). Notably, the trial was impacted by the COVID-19 pandemic. A pre-specified analysis censoring follow-up in late 2020 showed a statistically significant 24% relative risk reduction in the primary outcome with IV iron (rate ratio 0.76; *p* = 0.047) (59). No increase in infection-related hospitalizations or deaths was seen with IV iron, and fewer patients on IV iron experienced cardiac adverse events (59). IRONMAN supports the benefit of IV iron in reducing HF events, though the open-label design and pandemic interference tempered the results.

The HEART-FID trial was the largest IV iron trial to date (*n* = 3,065, multicenter) in HFrEF with iron deficiency. Patients were randomized to FCM or placebo, with repeat dosing over six months. The primary endpoint was a hierarchical composite of death, cardiovascular hospitalizations, and change in six-minute walk distance. At 12 months, the trial found no significant difference between the groups on the composite outcome (61). Numerically, 12-month mortality was slightly lower with FCM (8.6% vs. 10.3%), and fewer total HF hospitalizations occurred (297 vs. 332), but these differences did not translate into a statistically significant clinical benefit. The change in six-minute walk distance at six months was modest (+8 m with FCM vs. +4 m placebo) (61). On the positive side, repeated IV iron was confirmed to be safe, with similar rates of adverse events between FCM and placebo (serious adverse events ∼27% in both) (61). HEART-FID's neutral result has prompted further analysis, including pooling data from trials.

IV iron therapies (FCM and ferric derisomaltose in these trials) have shown a good safety profile in HF. Infusion reactions or anaphylaxis are rare with modern formulations. Across trials, rates of adverse events, including infections, were comparable between iron and placebo groups (59, 61). In fact, IRONMAN reported no increase in infections and even fewer serious cardiac adverse events with IV iron vs. standard care (59). Aside from infusion reactions, IV iron preparations have been associated with a rapid increase in NTBI, which can form hydroxyl radicals, leading to oxidative injury in tissues via the Fenton reaction (62). Transient hypophosphatemia can occur with high-dose FCM, but clinical sequelae are uncommon in the short term. Overall, patients receiving IV iron often report improved well-being and exercise tolerance within weeks of treatment (57). Proper patient selection and monitoring (iron indices, hemoglobin, etc.) are important to maximize benefit and avoid unnecessary treatment in those without true iron deficiency.

While IV iron has indisputably improved symptoms and exercise capacity in iron-deficient HF, its impact on long-term “hard” outcomes (mortality and major cardiovascular events) remains an area of active investigation. For example, trials like HEART-FID, despite numerical improvement, did not achieve statistical significance in reducing all-cause mortality or HF hospitalizations. No single trial to date has definitively proven a mortality benefit. Meta-analyses of RCTs (including FAIR-HF, CONFIRM-HF, EFFECT-HF, AFFIRM-AHF, and IRONMAN) consistently show a reduction in HF hospitalizations with IV iron and suggest a trend toward improved survival (60, 63), but results of large outcomes trials have been mixed. This inconsistency has led to debate on optimal timing and patient selection for IV iron therapy. For instance, some analyses indicate patients with more severe iron deficiency (transferrin saturation <20%) derive the greatest benefit (63), whereas those with only borderline low ferritin might see less impact. The dosing strategy (single repletion vs. intermittent maintenance infusions) and the specific formulation could also influence outcomes, though no head-to-head trials of iron preparations have been done in HF.

Current HF guidelines (ESC and ACC/AHA) have incorporated IV iron as a class IIa recommendation for HFrEF patients with iron deficiency, to improve exercise tolerance and reduce the risk of hospitalization. Ongoing studies and longer-term follow-up may clarify if IV iron can confer mortality benefits or if certain subgroups (e.g., those post-acute HF or with advanced symptoms) should be prioritized. Despite some uncertainties, intravenous iron has become an important adjunct therapy in HFrEF over the past decade, addressing a modifiable co-morbidity that contributes to patients' symptoms and clinical outcomes (61). The therapy exemplifies a move toward treating systemic contributors to HF (like iron deficiency) in addition to direct cardiac targets, with the ultimate goal of improving both the quality and quantity of life for these patients.

### 3.4 Anti-fibrotic agents

Myocardial fibrosis (MF) is present in all patients with chronic heart failure and is characterized by collagen deposition and abnormal distribution of extracellular matrix fibroblasts (64). It can result both due to reactive processes or aging, as well as following cardiomyocyte injury and death (65). Growth factors and pro-inflammatory cytokines involved in myocardial fibrosis include connective tissue growth factor (CTGF), tissue growth factor-β (TGF-β), tumour necrosis factor-α (TNF-α), interleukin-11 (IL-11) and galectin-3 (Gal-3). However, RAAS can also promote MF (66).

The use of antifibrotic agents in HF patients has gained ground. In 2021, the PIROUETTE trial evaluated the use of pirfenidone in HFpEF patients and found that compared to placebo, pirfenidone was able to reduce the myocardial extracellular volume (ECV) by 0.7%, without affecting patients' diastolic function and 6-minute walk distance (67). This difference is significant, given that even a 1% increase in ECV is associated with greater all-cause mortality in patients with MF (68). Normal ECV values range between 20% and 26%, with increased values usually observed in dilated and hypertrophic cardiomyopathies (69). It can be approximated by using T1 cardiac magnetic resonance imaging (MRI), as well as by cardiac computed tomography (CT) in individuals with contraindications to the former.

Although multiple trials have evaluated the use of anti-fibrotic therapies in conditions such as diabetes mellitus, hypertension, and cardiac sarcoidosis, current trials on MF involving patients with HF primarily revolve around GDMT agents (70). A randomized controlled trial by McDiarmid et al. evaluating spironolactone use in HFpEF subjects found no significant change in ECV compared to placebo, despite the presence of a significant decrease in the rate of extracellular expansion (71). Similar findings were reported in the study by Mizutani et al., in which the use of Entresto in HFrEF subjects did not result in ECV changes, but did result in decreases in both extra- and intracellular mass (72). Nonetheless, use of ARNIs and MRAs has been shown to attenuate the levels of fibrosis related markers, including procollagen type I C-terminal propertied (PICP) and Procollagen III N-terminal peptide (PIINP) (70).

A study by Requena-Ibáñez et al. found that the use of Empagliflozin, an SGLT2i in nondiabetic patients with HFrEF, resulted in a reduction in extracellular volume, including both matrix and cardiomyocyte volume (73). The ongoing CARDIA-STIFF study (NCT04739215) will aim to evaluate the effects of Dapagliflozin in HFpEF by measuring changes in serum levels of PICP and collagen type I C-terminal telopeptide to matrix metalloproteinase ratio (CITP:MMP-1),which are biomarkers of collagen type I deposition and the degree of collagen type cross-linking respectively (74).

Finally, multiple other antifibrotic agents have been described in literature. These include colchicine, which is known to alleviate cardiac fibrosis and diastolic dysfunction by reducing the expression of the NLR family pyrin domain containing 3 (NLRP3) pathway, TGF-β inhibitors such as metelimumab and fresolimumab, Platelet-Derived Growth Factor (PDGF) inhibitors such as pegpleranib, and inhibitors of the Rho-associated coiled-coil forming kinases ROCK1 and ROCK2, the latter of which are serine/threonine kinases involved in actin cytoskeleton remodeling (75). Colchicine improves diastolic function in rodent models of HFpEF and is currently under investigation in a clinical trial of HFpEF patients (76). TGF-beta inhibitors have not yet been evaluated in heart failure models; however, they have shown promise in fibrotic pathologies such as systemic sclerosis and idiopathic pulmonary fibrosis. Similarly, pegpleranib is only currently in use for age-related macular degeneration, and therefore any theoretical benefit in myocardial fibrosis is purely speculative—mechanistically, however, PDGF blockade does appear to be an attractive target for ameliorating cardiac fibrosis, as evidenced by studies in rodent models such as demonstrably reducing atrial fibrosis in a model of pressure-overloaded mouse hearts (77). Alternatively, the rho kinase inhibitor fasudil has been shown to improve hemodynamic parameters in a human study of pulmonary hypertension HFpEF, but true patient-centered outcome data has yet to be collected, thus limiting clinical applicability at this time (78). It is important to note that the aforementioned non-GDMT antifibrotic agents are still under investigation, and therefore their clinical efficacy at this time is yet to be established; nonetheless, these agents provide promising and mechanistically intriguing pathways for future development and research. Thus, future trials could investigate the use of these agents in HF patients who carry the burden of MF.

### 3.5 Stem cell-based therapies

There has been an increase in interest in stem cell therapies due to their potential to improve heart function and reduce cardiac remodeling (79). They do so through the secretion of various factors. These include the fibroblast growth factor 2 (FGF-2), which reduces ischemia-induced myocardial apoptosis through the increased expression of the anti-apoptotic protein Bcl-2, as well as secreted factors angiopoietin-1 (Ang-1), angiopoietin-2 (Ang-2) and vascular endothelial growth factor (VEGF) which have been shown to increase vascular density and blood flow in the ischemic heart (80). A meta-analysis by Kavousi et al. found that patients with symptoms of HF who received transplantation of mesenchymal stem cells (MSCs) through either vessels (either intracoronary or intravenous) or direct injection to the myocardium had decreased mortality and improved LVEF and NYHA classification (81). These MSCs were obtained from various sources, including umbilical cord, bone marrow, and adipose tissue. Stem cells obtained from these lines have shown promise, unlike human embryonic stem cells (hESCs), the latter of which have been shown to disappear rapidly after transplantation and to be more prone to graft rejection (82). Similarly, challenges have been presented with induced pluripotent stem cell (iPSC)-derived cardiomyocytes (CMs); although iPSCs are believed to exhibit similar self-renewability to ESCs, iPSC-derived CMs have largely failed to function as adult CMs (83). There have been clinical trials underway to investigate the efficacy and safety of many of the aforementioned stem cell methods. Phase 3 DREAM-HF showed that a one-time trans-endocardial injection of rexlemestrocel-L allogeneic mesenchymal precursor cells in 537 HFrEF patients enhanced LVEF at 12 months and decreased CV death/MI/stroke by ≈37% at 30 months within the high-inflammation subgroup, although the trial did not meet its primary HF-hospitalization endpoint (84). In HFpEF, small crossover CELL-pEF pilot (30 patients) showed autologous CD34^+^ cell injections decreased E/e′ by ≈3.8, lowered NT-proBNP and improved 6-min-walk distance at 6 months (85). In HFrEF, randomized RIMECARD (*n* = 30) identified intravenous umbilical-cord MSCs as safe and improved LVEF +7% vs. +1.9% with placebo at one year, with additional NYHA and quality-of-life improvement (Safety and Efficacy of the Intravenous Infusion of Umbilical Cord Mesenchymal Stem Cells in Patients With Heart Failure: A Phase 1/2 Randomized Controlled Trial (RIMECARD Trial [Randomized Clinical Trial of Intravenous Infusion Umbilical Cord Mesenchymal Stem Cells on Cardiopathy]) - PubMed); a 2023 meta-analysis of three UC-MSC RCTs confirmed a pooled +3.2% EF benefit without mortality decrement (The Safety and Efficacy of Human Umbilical Cord-Derived Mesenchymal Stem Cells in Patients With Heart Failure and Myocardial Infarction: A Meta-Analysis of Clinical Trials - PubMed). Allogeneic cardiosphere-derived cells also look promising: the DYNAMIC Phase I/II trial in dilated-CM (14 patients) increased EF from 22.9 to 26.8% and reduced LV volumes at 6 months, steering bigger trials such as REGRESS-HFpEF (CAP-1002), whose results are not yet out (86).

In late 2024, Zhang et al. published a list of recent and ongoing clinical trials involving stem cell therapy for HF (87). The documented trials did record the presence of improvement in clinical outcomes, however, adverse events such as death, MIs, and arrhythmias were recorded as well. Indeed, although stem cell therapies raise hope for a promising treatment of ischemic heart disease, there are still barriers in regards to both ethical concerns and low cell survival and engraftment rates (88). For this reason, scientists will be tasked with addressing variability in outcomes and raising ethical questions, such as concerns regarding genetic modifications of cells and protecting the confidentiality of cell donors (89).

### 3.6 RNA-based therapies

RNA-based therapies also exist, including antisense oligonucleotides (ASOs) and small-interfering RNAs (siRNAs). As far as ASOs are concerned, they are short single single-stranded, synthetic RNA or DNA molecules that bind to specific RNA sequences to either promote RNA cleavage or interfere with mRNA translation or maturation (90). By doing so, they block the production of an abnormal target protein or stimulate the production of a normal one. An example of an ASO drug that is involved in reducing the cardiovascular disease burden is mipomersen. Mipomersen targets hepatic apolipoprotein-B-100 mRNA and reduces apoB-100 secretion, which further reduces low-density lipoprotein cholesterol (LDL-C) secretion and levels of lipoprotein (a) (Lp(a)) (91, 92). Phospholamban ASOs (PLN-ASOs) are being studied. PLN is an inhibitor of the sarcoplasmic reticulum Ca^2+^-ATPase (SERCA2a), which is involved in maintaining appropriate sarcoplasmic reticulum Ca^2+^ cycling for cardiac relaxation and contraction (93). PLN- ASOs thus reduce *Pln* mRNA expression and PLN protein production to prevent and reverse cardiac dysfunction. Triantennary N-acetylgalactosamine (GalNAc3) and GalNAc3-conjugated 2'-O-methoxyethyl (2'MOE) modiﬁed ASOs are also ASOs of interest that are currently being evaluated in ongoing clinical trials (19). They also target RNA expressed by hepatocytes, aiming to reduce elevated Lp(a) levels. Fesomersen and eplontersen are examples of GalNAc ASOs, while inotersen is an example of a 2'-MOE ASO (92).

Unlike ASOs, siRNAs are double-stranded RNA molecules that bind to a specific mRNA sequence to create an RNA-induced silencing complex (RISC) that is responsible for the cleavage and degradation of the target mRNA (94). One of the most well-known siRNAs is inclisiran, a proprotein convertase subtilisin/kexin type 9 (PCSK9) mRNA targeting siRNA (95). PCSK9, a plasma protein that participates in the degradation of LDL receptor levels, plays a crucial role in the progression of atherosclerotic disease by increasing the levels of LDL cholesterol in the plasma (96). Other important siRNA molecules include ARO-ANG3, which targets angiopoietin-like 3 (ANGPTL3), a protein that inhibits the lipoprotein lipase and thus the clearance of triglyceride-rich lipoproteins, and plozasiran, an inhibitor of apolipoprotein C3 (ApoC3), which is known for also having lipid-lowering effects (95). To uncover more of the beneficial effects of siRNAs, ongoing clinical trials involving siRNAs involve those studying zerlasiran and lepodisiran, siRNA molecules which target Lp(a) (97, 98).

The upcoming importance of microRNAs (miRNAs) is important. There are thousands of unique miRNAs in the human body, which are mostly divided into two categories that depend on whether they have pro-atherosclerotic or anti-atherosclerotic properties (99). For example, miR-21 has been shown to downregulate the angiotensin-converting enzyme 2 (ACE2), an enzyme that protects against the blood pressure-raising effects of angiotensin II (100). Studies targeting this enzyme have shown reductions in both blood pressure and vascular inﬂammation. On the other hand, miR-126 has been shown to promote endothelial proliferation and inhibit atherosclerotic lesion progression (101). For this reason, novel therapies need to either inhibit miRNAs with proinflammatory and pro-atherosclerotic properties or stimulate miRNAs that aim to reduce oxidative stress and cardiomyocyte damage. Moreover, long non-coding RNA (lncRNA) therapies like Hotair and Malat1 are other potential RNA therapies that, similar to miRNAs, could help induce revascularization, but their function is largely unknown (102). At the time of writing, the clinical applicability of RNA therapies is limited as they are still largely under investigation.

### 3.7 Gene-based therapies

Gene therapy has gained ground in recent years. It employs the delivery of exogenous genes to supplement insufficient protein levels (103). These insufficient protein levels result when the structure and function of gene-encoded proteins have been altered by pathogenic variants (PVs). One of the main mechanisms for the delivery of gene therapies is using viral vectors, such as recombinant adeno-associated viruses (AAVs), due to their ability to integrate their genetic material into cells of a host organism (104). Although AAV vectors can still generate neutralising anti-AAV antibodies, their lack of immunogenicity, as well as their numerous AAV serotypes, make them a favorable type of gene therapy.

Other vectors commonly used for gene therapies for HF include naked plasmid DNA and adenovirus. Delivery of adenoviral-mediated SERCA2a-DNA has been shown to greatly enhance Ca^2+^ handling in human CMs and LV systolic and diastolic performance (105). Like AAVs, plasmids, which are made of circular, double-stranded DNA, have little immunogenicity and can propagate in large quantities due to their large DNA packaging capacity (106). Nonetheless, they possess limited gene transfer abilities.

Scientists from the University of Utah studying the cardiac bridging integrator 1 (cBIN1), a transverse-tubule scaffolding protein that is involved in calcium, found that a low dose of cBIN1 delivered in minipigs using AAV9 (an AAV serotype), was associated with reduced pulmonary and systemic fluid retention and increased survival (107). Although it may be hard to extrapolate findings from animal studies to humans, there are currently ongoing trials involving gene therapies. For example, GenePHIT, is an ongoing trial that is evaluating an AAV-based gene therapy known by the name AB-1002 in patients with nonischemic cardiomyopathy and NYHA Class III (108). AB-1002 aims to increase the production of protein inhibitor 1 (I-1c) and thus decrease the action of protein phosphatase 1, a protein linked to HF. Similarly, another AAV-based gene therapy trial is evaluating SRD-001, a gene therapy that is delivered directly to cardiac ventricular muscle cells to increase the functional activity of SERCA2a (109). This trial, however, includes patients with ischemic cardiomyopathy. It will thus not be surprising if more up-and-coming gene therapies enter the market. Their appealing benefits will expedite their usage to purposes outside of HF, especially taking into consideration the Cleveland Clinic's recent in-human gene therapy for hypertrophic cardiomyopathy (110). It is, however, important to note that the gene therapies discussed above are, at the time of writing, purely investigational and therefore while representing a new frontier of possible treatment modalities are, as of yet, not applicable in the clinical setting.

## 4 Current guidelines

In 2023, the European Society of Cardiology (ESC) published updates regarding the management of heart failure. As far as the management of patients with symptomatic HFpEF is concerned, the most recent guidelines recommend diuretics for water retention, SGLT-2 inhibitors such as Dapagliflozin and Empagliflozin and addressing any cardiovascular and non-cardiovascular comorbidities (111). In addition to the use of diuretics and SGLT-2 inhibitors, the ESC guidelines recommend the use of ACEi/ARNI/ARB, MRAs and beta blockers in patients with HFmrEF, while also emphasizing the use of finerenone and SGLT-2 inhibitors recommended to prevent HF and reduce the risk of HF hospitalization in patients with CKD and type 2 diabetes. As far as the management of patients with HFrEF is concerned, it is identical to that of symptomatic patients with HFmrEF and unchanged from the 2021 guidelines, with great emphasis placed on the initiation and rapid up-titration of treatment before discharge for patients hospitalized for acute heart failure (111, 112). Moreover, the 2021 guidelines include the possible use of I_f_-channel inhibitors such as Ivabradine for the reduction of cardiovascular mortality and HF hospitalization in patients with symptomatic HFrEF with an LVEF <35%, with HF hospitalization in recent 12 months, in sinus rhythm (SR) and with a heart rate greater than 70 bpm (beats per minute), who are already on evidence-based therapy (MRA, ACEi/ARB, beta blockers) (112).

When it comes to the most recent 2022 AHA guidelines, the same treatment recommendations apply, along with up-titration of GDMT every 1–2 weeks in patients with HFrEF (1). They also state that for patients self-identified as African American with NYHA class III-IV HFrEF who are receiving optimal medical therapy, the combination of hydralazine and isosorbide dinitrate in addition to GDMT is recommended to improve symptoms and reduce morbidity and mortality. ESC guidelines uphold this but also suggest this medical combination in patients with HFrEF who cannot tolerate any of an ACE-I, ARNI, or an ARB (or if their use is contraindicated) to reduce mortality (112). Another AHA recommendation is that the use of MRAs and ARNI/ARBs in selected patients with HFpEF, particularly among patients with LVEF on the lower end of this spectrum, may be considered to decrease hospitalizations (1). Finally, although the 2022 AHA guidelines state that the use of digoxin may be considered to decrease HF hospitalizations in HFrEF patients who are on GDMT or who cannot tolerate it, ESC guidelines state that the use of digoxin in those with HFrEF and HFmrEF in SR has demonstrated no reduction in mortality (1, 112).

## 5 Challenges and limitations in treatments

Barriers in medical therapy for HF span both the currently established GDMT and novel therapies, such as gene therapies. Starting from conventional therapies, current existing guidelines do not specify the order in which individual GDMT components need to be given, nor the initiation timing or up-titration strategies (113). Furthermore, limited patient knowledge on the importance on adherence to GDMT, combined with limitations in assessing cardiovascular specialists and securing outpatient appointments for uptitration of the GDMT every 1–2 weeks, further complicates access to adequate patient care (114). As far as novel therapies are concerned, factors such as anatomical and biological difficulties for targeting cardiomyocytes, along with presented immunogenicity against administered vectors, play a role in observed responses (115). For example, gene therapies using adenovirus have been shown to induce potent CD8+ T-cell immune responses, while AAV-based therapies have also been reported to induce T-cell-mediated immune responses resulting in liver injury.

For those receiving treatment, although the use of ARBs and ACEi has shown a reduction in mortality in patients with HFrEF. In patients with HFpEF, trials have not demonstrated efficacy of RAAS blockade added to GDMT (116). Thus, true molecular reversal of many of the underlying HF mechanisms is still an elusive goal. Moreover, when it comes to hospitalized patients with cardiovascular disease, factors according to Niriayo et al., identified to predispose to drug therapy problems include older age and polypharmacy, the latter of which is considered to increase the risk of adverse drug reactions, drug interactions, medication non-adherence, and medication errors (117). For this reason, HF management should remain individualized, evaluating patient characteristics, comorbidities, and preferences (118).

## 6 Conclusion

In summary, various novel therapies have started to emerge outside of the GDMT classes that are commonly prescribed to HF patients. These include vericiguat, antifibrotic agents, as well as gene, RNA-based, and stem-cell therapies. Although promising results have been suggested for each type of therapy, social barriers in introducing and up-titrating therapies, lack of established guidelines on treatment options, and limitations associated with each therapy make HF management more challenging. However, like previously mentioned, treatment should remain individualized, taking into consideration patients' needs and preferences.
